# Pharmacological thromboprophylaxis and its impact on venous thromboembolism following total knee and hip arthroplasty in Korea: A nationwide population-based study

**DOI:** 10.1371/journal.pone.0178214

**Published:** 2017-05-24

**Authors:** Ho-Young Yhim, Juhyun Lee, Ji Yun Lee, Jeong-Ok Lee, Soo-Mee Bang

**Affiliations:** 1 Department of Internal Medicine, Chonbuk National University Medical School, Jeonju, Korea; 2 Research Institute of Clinical Medicine, Chonbuk National University-Biomedical Research Institute of Chonbuk National University Hospital, Jeonju, Korea; 3 Department of Internal Medicine, Seoul National University Bundang Hospital, Seongnam, Korea; Holbæk Hospital, DENMARK

## Abstract

**Background:**

Limited data is available regarding the pharmacological prophylaxis for venous thromboembolism (VTE) in Asian patients undergoing total knee arthroplasty or total hip arthroplasty (TKA/THA).

**Methods:**

We performed a population-based epidemiological study using the Health Insurance Review and Assessment Service database to estimate the rate of pharmacological thromboprophylaxis and its impact on VTE in Korean patients who underwent TKA/THA between 2009 and 2013.

**Results:**

We identified 306,912 cases (TKA, 261,260; THA, 45,652). The pharmacological thromboprophylaxis rate was 57.16% (TKA, 58.32%; THA, 50.51%), which increased from 42.81% in 2009 to 65.92% in 2013 (*P* = 0.0165). Both low-molecular-weight-heparin (22.42%) and rivaroxaban (22.71%) were the most common drugs for prophylaxis. The number of patients aged ≥ 60 years (87.31% *vs*. 81.01%, *P* < 0.0001), cases requiring general anesthesia (20.70% *vs*. 18.37%, *P* < 0.0001), and cases requiring long hospital stay (median, 13 days vs. 12 days, *P* < 0.0001) were significantly greater in the pharmacological prophylaxis group. The incidence of VTE within 3 months of surgery was 1.52% (TKA, 1.46%; THA, 1.87%). Patients with pharmacological prophylaxis had higher VTE rates (TKA, 1.69% *vs*. 1.14%; THA, 2.30% *vs*. 1.43%) than those without prophylaxis, with advanced age, use of general anesthesia, and a longer hospital stay increasing the risk of VTE. However, rivaroxaban significantly reduced the incidence of VTE following TKA (0.82% *vs*. 1.14%; odd ratio [OR], 0.72; 95% CI, 0.65–0.79). Moreover, ≥ 10 days of pharmacological thromboprophylaxis was significantly associated with lower incidence of VTE after TKA (1.33% *vs*. 1.52%; OR, 0.87; 95% CI, 0.81–0.94).

**Conclusion:**

This represents the largest epidemiological study showing a gradual increase in the use of pharmacological prophylaxis in Korean patients undergoing TKA/THA. Although the incidence of VTE is still low without pharmacological prophylaxis, this study demonstrates that the incidence of VTE can be reduced further using appropriate pharmacological thromboprophylaxis strategies.

## Introduction

Venous thromboembolism (VTE), consisting of deep venous thrombosis (DVT) and pulmonary embolism (PE), is a common complication following total knee arthroplasty or total hip arthroplasty (TKA/THA) [1]. Because patients undergoing TKA/THA are considered to be at high risk for developing VTE in comparison with patients undergoing other surgical procedures, the international guidelines recommend pharmacological prophylaxis as a routine strategy for those without high risk for bleeding [1,2]. However, these international guidelines are based on data predominantly derived from studies conducted in Western populations.

In general, the incidence of VTE after TKA/THA in Asian populations has been lower than that in Western populations [3–5]. The SMART study, an international prospective study on Asian orthopedic patients, showed that the incidence of symptomatic VTE or sudden death without thromboprophylaxis was 1.5% within 1 month of a major orthopedic surgery [6], which was approximately one third of the incidence estimated in Western populations [1]. Our previous population-based epidemiological study also showed that the incidences of VTE in Korean patients were 1.08% and 0.98% within 5 weeks of TKA and THA, respectively [3]. Moreover, because of increased risk of bleeding complications associated with anticoagulant use, orthopedic surgeons may have concerns regarding the prophylactic use of these drugs [7,8]. Therefore, when using pharmacological prophylaxis, it is important to recognize the fine balance between VTE prevention and associated adverse events from drug use, particularly in the low incidence population [9]. However, due to the lack of specific data regarding its value on low incidence population, there is some debate whether international guidelines are appropriate for the management of Asian populations undergoing TKA/THA [10].

Until recently, a considerable number of Asian hospitals did not incorporate pharmacological prophylaxis as part of the standard care for patients undergoing TKA/THA [10,11]. In a Taiwanese population-based study, the overall pharmacological prophylaxis rate after TKA was only 2.2% [4]. However, the incidence of DVT in Asian populations was comparable with that in Western populations, approaching approximately 40%, in other studies regarding venographically defined DVT following major orthopedic surgery [12,13]. Such results may be explained by the rapid growth of elderly populations and the westernization of lifestyle in Asian countries [14,15]. As such, the awareness of VTE risks in Asian populations has been gradually increasing, which has led to a reconsideration of appropriate pharmacological prophylaxis for VTE after major orthopedic surgery in Asian populations. Although the risk of postoperative VTE varies considerably between Asian populations, the Korean population is well known to have a relatively low risk for VTE after major orthopedic surgery. In the AIDA study [13], the venographic DVT rate in the Korean population (29.8%) was lower than that in the Chinese (45.0%) or in non-Korean-non-Chinese populations (46.3%). Particularly, the rate of symptomatic VTE was just approximately 1% after major orthopedic surgery in the prior Korean epidemiologic study [3]. Thus, the Korean epidemiologic data regarding the pharmacological prophylaxis strategy after TKA/THA may help other Asian populations with a relatively lower risk to estimate benefit from this preventive strategy.

We conducted a population-based epidemiological study using the Health Insurance Review and Assessment Service (HIRA) database in Korea. This study was designed to assess the proportion and recent trends of patients who received pharmacological prophylaxis after TKA/THA, and to evaluate the clinical impact of pharmacological prophylaxis on postoperative VTE and bleeding complications in the Korean population.

## Materials and methods

### Data sources and study cohort

This population-based study was conducted using the HIRA database. HIRA is a government-affiliated organization building an accurate claims review and quality assessment system for the National Health Insurance (NHI) and Medical Aid programs. The features of the database in the epidemiological studies regarding VTE have already been described in previous studies [3,14,16]. Korea has been operating the NHI program, which is an obligatory social insurance system, covering approximately 97% of the entire population [17]. The rest of the population, approximately 3%, is covered under the Medical Aid program, which complements the NHI to provide effective medical services for low-income households. In brief, if patients are under NHI program, patients pay an average of 30% of the total medical cost when they use the hospitals. Subsequently, the hospitals submit inpatients and outpatients claims data to the HIRA for reimbursement from the government of the remaining 70% of the total cost. In contrast, the patients do not pay for hospitals if they belonged to the Medical Aid program. Instead, the government pays the full medical costs after reviewing the claims data from hospitals. The claims data from both NHI and Medical Aid programs were processed through the Electronic Data Interchange (EDI) system and reviewed by the HIRA. Thus, the HIRA database contains virtually all hospital-based information for payments in all Korean residents. Actually, based on data from the Korean National Statistical Office (http://kosis.kr), the Korean population was 51,245,707 in 2016, and the number of registered people in the NHI was 52,272,755 in 2016. Although a small difference in the number of people between the Korean National Statistical Office and NHI is present due to overseas Koreans, conducting epidemiological study using the HIRA database in Korean population is possible. The HIRA database provides inpatient and outpatient data, including demographic information, date of hospital visit, all prescription drugs during inpatient and outpatient visits, hospital admissions, procedures including operations, and transfusion information. Drug information included the name, drug code, prescription date, dose, duration and route of drug administration. The study cohort comprised of all patients who underwent TKA or THA with a record in the HIRA database between January 1, 2009 and December 31, 2013. In cases with bilateral surgery, cases were considered as a single patient if second surgery was not performed after 3 months of the first surgery. In this study, however, we excluded patients who underwent hip fracture surgery to assess the status of pharmacological prophylaxis in an elective setting of major orthopedic surgery. This study was approved by the Institutional Review Board of Seoul National University Bundang Hospital, which waived the requirement for informed consent.

### Case identification

A case was considered VTE if it concurrently satisfied both diagnostic and drug codes for VTE within 90 days of surgery. The VTE codes in the fifth edition of the Korean Classification of Disease (KCD-5) diagnostic codes, which was a modified version of the International Classification of Disease (ICD-10), included DVT (I80.2, I80.3) and PE (I26, I26.0, I26.9). Simultaneously, the EDI drug codes for unfractionated heparin (UFH), low-molecular-weight-heparin (LMWH), and rivaroxaban (≥ 15 mg/day), which were unique 9-digit numeric and alphabetic identifiers assigned to each medicine in the HIRA database, were also needed for a case to be considered as VTE. Other direct oral anticoagulants, except rivaroxaban (i.e. dabigatran, apixaban, and edoxaban) and fondaparinux were not included in drug codes for VTE, because these were not approved to treat VTE in Korea during the study period. Based on this definition, the VTE cases identified in this study might be able to be considered as having symptomatic events because the postoperative routine surveillance for VTE and the treatment was not recommended in asymptomatic patients [3]. However, it is likely that some fatal PE cases could not be identified as VTE cases if drugs were not administered.

In this study, four drugs (fondaparinux, rivaroxaban [10 mg/day], LMWH, and aspirin) were investigated with respect to the use in prophylaxis after TKA/THA. Pharmacological thromboprophylaxis was considered when the first dose of these four drugs was administered within 3 days of surgery. However, LMWH can be used to treat the VTE based on the body weight of patients, and personal information, like body weight, was not included in the HIRA database. In addition, the doses for thromboprophylaxis in each LMWH were heterogeneous across the institutions in Korea. As such, escalating the dose of LMWH or changing to other drugs for VTE treatment within 90 days of surgery were considered to define the pharmacologic prophylaxis using LMWH in patients with VTE. In addition, with respect to aspirin, pharmacological prophylaxis was considered if aspirin was maintained for a maximum of 42 days to exclude long-term aspirin users for other indications (i.e. cardiovascular disease). Warfarin and low-dose UFH were rarely used for VTE prophylaxis following TKA/THA in Korea due to its inconvenience. Therefore, warfarin and low-dose UFH were not included in this investigation.

### Statistical analysis

The primary study outcome was the rates and annual trends of pharmacological prophylaxis in Korean patients undergoing TKA/THA between 2009 and 2013. The secondary outcomes were the rates of pharmacological prophylaxis ≥ 10 days, and the incidence of VTE and the amount of transfused red blood cells based on the pharmacological prophylaxis status. The postoperative bleeding outcome was indirectly estimated using the amount of transfused red blood cells during the hospital stay after surgery. The number of TKA/THA was evaluated by age, sex, type and length of pharmacological prophylaxis, and mode of anesthesia, and was compared using χ^2^ test for categorical variables and Mann-Whitney test for continuous variables. The rates of prophylaxis and the incidence of VTE were estimated as the number of appropriate events per 100 cases for TKA/THA and were expressed as a percentage (%) with corresponding 95% confidence interval (CI) and compared using χ^2^ test. The annual percentage change (APC), which was determined using a log-linear regression model, was used to investigate the trends for pharmacological prophylaxis over the study period. A multivariate logistic regression analysis was performed to find independent risk factors for VTE development from clinical variables, and the results were reported with an odds ratio (OR) and 95% CI. A *P* < 0.05 was considered to be statistically significant for all analyses. Statistical analyses were performed using SAS Enterprise Guide 6.4 (SAS institute Inc., Cary, NC, USA).

## Results

### Study cohort and pharmacological prophylaxis following TKA/THA

For 5 years, 306,912 patients who underwent TKA/THA were identified in the HIRA database. Of these, 261,260 patients (85.13%) underwent TKA and the remaining 45,652 patients (14.87%) underwent THA. Bilateral joint arthroplasty was performed in 12,700 (4.86%) and 1,488 (3.26%) patients with TKA and THA, respectively. The clinical characteristics of the study cohort are listed in Table 1. When we compared age, duration of hospitalization, and mode of anesthesia based on the pharmacological prophylaxis status, the number of patients aged ≥ 60 years were significantly greater in patients with pharmacological prophylaxis (87.31% *vs*. 81.01%; *P* < 0.0001), than in those without prophylaxis. In addition, patients with general anesthesia (20.70% *vs*. 18.37%; *P* < 0.0001) and long hospital stay (median, 13 days *vs*. 12 days; *P* < 0.0001) received more pharmacological prophylaxis. Thus, patients that were expected to have a higher risk for postoperative VTE received more pharmacological prophylaxis in our cohort.

**Table 1:** Clinical characteristics of the study cohort.

	All patients (No., %)	Pharmacological prophylaxis (No., %)	No pharmacological prophylaxis (No., %)	*P* value*
No. of patients	306,912	175,430	131,482	
Surgery				
TKA	261,260 (85.13)	152,373 (86.86)	108,887 (82.82)	< 0.0001
THA	45,652 (14.87)	23,057 (13.14)	22,595 (17.18)	
Age				
< 60 years	47,228 (15.39)	22,263 (12.69)	24,965 (18.99)	< 0.0001
≥ 60 years	259,684 (84.61)	153,167 (87.31)	106,517 (81.01)	
Sex				
Male	58,136 (18.94)	30,755 (17.53)	27,381 (20.82)	< 0.0001
Female	248,776 (81.06)	144,675 (82.47)	104,101 (79.18)	
Duration of hospitalization				
Median (range)	12 (1–370)	13 (1–370)	12 (1–204)	< 0.0001
Mode of anesthesia				
Regional anesthesia	244,002 (80.14)	139,115 (79.30)	107,331 (81.63)	< 0.0001
General anesthesia	60,466 (19.86)	36,315 (20.70)	24,151 (18.37)	

*P value indicates the comparison between the pharmacological and no pharmacological prophylaxis groups.

The rates of overall pharmacological prophylaxis in patients who underwent TKA/THA were 57.16% (Table 2). Both rivaroxaban (22.71%) and LMWH (22.43%) were the most common drugs used. The median duration of pharmacological prophylaxis was 9 days, and only 28.18% of patients received pharmacological prophylaxis ≥ 10 days (Table 2). The rates of pharmacological prophylaxis (58.32% *vs*. 51.51%; *P* < 0.0001) and ≥ 10 days of pharmacological prophylaxis (30.18% vs. 16.78%; *P* < 0.0001) in the TKA cohort were significantly higher than those in the THA cohort.

**Table 2:** Rates and types of overall and ≥ 10 days of pharmacological thromboprophylaxis between 2009 and 2013.

	All patients (No., %)	TKA (No., %)	THA (No., %)	*P* value
No. of patients	306,912	261,260	45,652	
Overall pharmacological prophylaxis	175,430 (57.16)	152,373 (58.32)	23,057 (50.51)	< 0.0001
Drugs for overall pharmacological prophylaxis				
Aspirin	28,176 (9.18)	24,612 (9.42)	3,564 (7.81)	
LMWH	68,834 (22.42)	55,181 (21.12)	13,653 (29.91)	
Rivaroxaban	69,702 (22.71)	64,859 (24.83)	4,843 (1.61)	
Fondaparinux	8,718 (2.84)	7,721 (2.95)	997 (2.18)	
Pharmacological prophylaxis ≥10 days	86,501 (28.18)	78,842 (30.18)	7,659 (16.78)	< 0.0001
Drugs for pharmacological prophylaxis ≥ 10 days				
Aspirin	14,820 (4.83)	12,812 (4.90)	2,008 (4.40)	
LMWH	15,730 (5.13)	13,791 (5.28)	1,939 (4.25)	
Rivaroxaban	53,605 (17.47)	50,085 (19.17)	3,520 (7.71)	
Fondaparinux	2,346 (0.76)	2,154 (0.82)	192 (0.42)	

### Annual trends of prophylaxis and VTE rates

**Fig 1:**
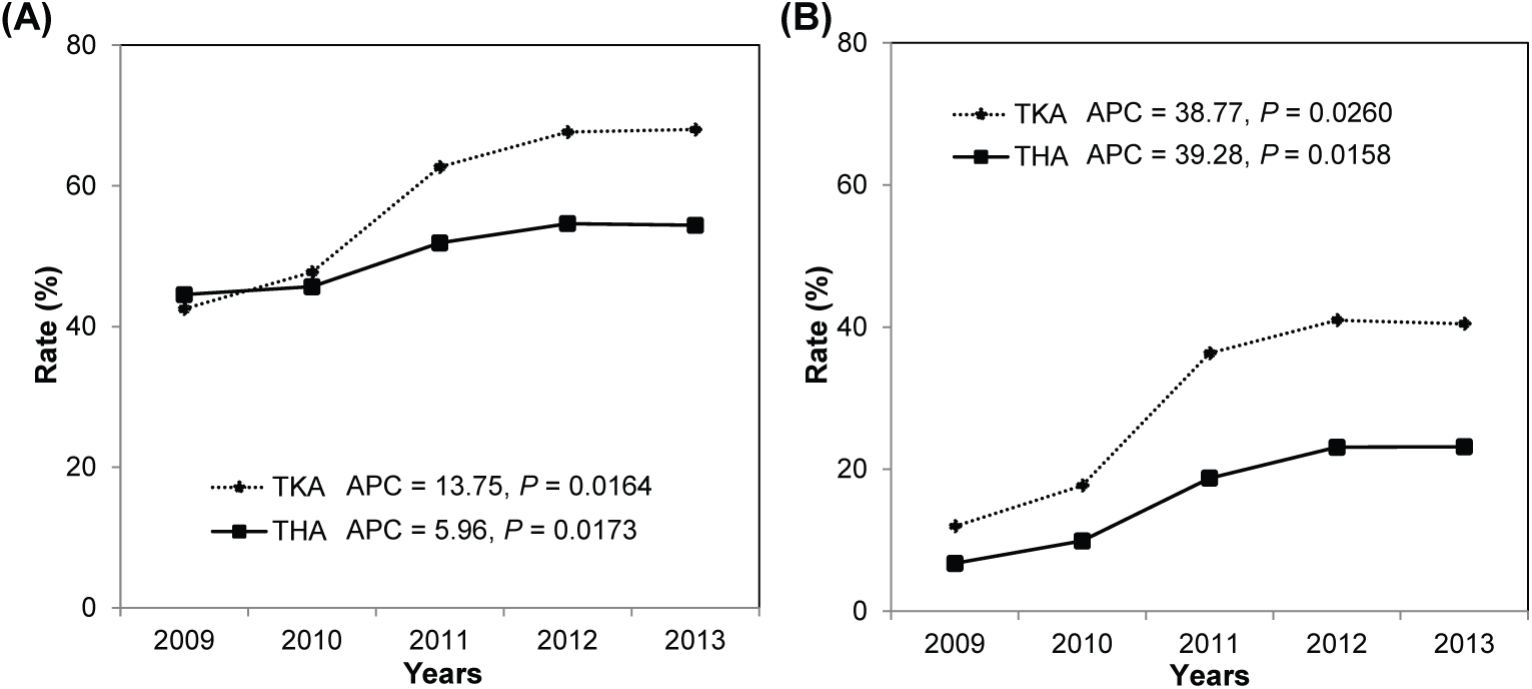
**Annual trends of the rates of (A) pharmacological prophylaxis and (B)** ≥ **10 days of pharmacological prophylaxis in patients who underwent TKA or THA between 2009 and 2013.** Abbreviations: TKA, total knee arthroplasty; THA, total hip arthroplasty; APC, annual percentage change

The rate of pharmacological prophylaxis increased by 12.64% annually from 2009 to 2013 (42.81% in 2009, 47.42% in 2010, 61.08% in 2011, 65.73% in 2012, and 65.92% in 2013), which was statistically significant (*P* = 0.0165). This trend was consistently observed in both TKA (APC, 13.75; *P* = 0.0164) and THA (APC, 5.96; *P* = 0.0173; Fig 1A) cohorts. The proportion of pharmacological prophylaxis ≥ 10 days also showed a marked increase in TKA (APC, 38.77; *P* = 0.0260) and THA (APC, 39.28; *P* = 0.0158; Fig 1B) cohorts. In contrast, the overall incidence of VTE following TKA/THA was not decreased during the 5-year study period (APC, 4.90; *P* = 0.2743).

### Association between VTE and pharmacological prophylaxis status

The incidence of VTE, DVT alone, and PE (with or without DVT) within 3 months of TKA/THA was 1.52% (N = 4,673), 0.94% (N = 2,901), and 0.58% (N = 1,772), respectively. The rates of VTE, DVT alone, and PE (with or without DVT) after each surgery are shown in Table 3.

**Table 3:** The incidence of venous thromboembolism within 3 months of TKA/THA between 2009 and 2013.

Type of surgery	No. of patients	No. of VTE	VTE rate (%, 95% CI)	No. of DVT alone	DVT alone rate (%, 95% CI)	No. of PE ± DVT	PE ± DVT rate (%, 95% CI)
TKA	261,260	3,820	1.46 (1.42–1.51)	2,376	0.91 (0.87–0.95)	1,444	0.55 (0.52–0.58)
THA	45,652	853	1.87 (1.75–2.00)	525	1.15 (1.06–1.25)	328	0.72 (0.64–0.80)
Total	306,912	4673	1.52 (1.15–1.57)	2,901	0.94 (0.91–0.98)	1,772	0.58 (0.55–0.60)

We compared the incidence of VTE in accordance with the pharmacological prophylaxis status to investigate the impact of pharmacological prophylaxis on postoperative VTE. Surprisingly, the present analysis showed that patients with pharmacological prophylaxis (i.e. aspirin, LMWH, rivaroxaban, fondaparinux) had significantly higher rates of postoperative VTE within 3 months than those without prophylaxis (1.77% *vs*. 1.19%; OR 1.50 [95% CI, 1.41–1.60]; Table 4). The incidences of VTE after each surgery were similar in the entire group.

**Table 4:** Incidence of venous thromboembolism based on the pharmacological thromboprophylaxis status.

Type of surgery	Pharmacological prophylaxis	No. of patients	No. of VTE	VTE rate (%, 95% CI)	OR (95% CI)	DVT alone rate (%, 95% CI)	OR (95% CI)	PE ± DVT rate (%, 95% CI)	OR (95% CI)
Any prophylaxis (aspirin, LMWH, rivaroxaban, fondaparinux; N = 175,430)									
Whole cohort	No	131,482	1,562	1.19 (1.13–1.25)	1	0.76 (0.71–0.81)	1	0.43 (0.39–0.46)	1
	Yes	175,430	3,111	1.77 (1.71–1.84)	1.50 (1.41–1.60)	1.08 (1.03–1.13)	1.43 (1.32–1.54)	0.69 (0.65–0.73)	1.62 (1.49–1.79)
TKA	No	108,887	1,240	1.14 (1.08–1.20)	1	0.74 (0.69–0.79)	1	0.40 (0.36–0.44)	1
	Yes	152,373	2,580	1.69 (1.63–1.76)	1.50 (1.40–1.60)	1.03 (0.98–1.08)	1.40 (1.29–1.53)	0.66 (0.62–0.70)	1.65 (1.48–1.85)
THA	No	22,595	322	1.43 (1.27–1.58)	1	0.88 (0.75–1.00)	1	0.55 (0.45–0.65)	1
	Yes	23,057	531	2.30 (2.11–2.50)	1.63 (1.42–1.87)	1.42 (1.27–1.57)	1.63 (1.36–1.94)	0.88 (0.76–1.01)	1.62 (1.29–2.02)
Aspirin (N = 28,176)									
TKA	No	108,887	1,240	1.14 (1.08–1.20)	1	0.74 (0.69–0.79)	1	0.40 (0.36–0.44)	1
	Aspirin	24,612	384	1.56 (1.14–1.72)	1.38 (1.23–1.54)	0.77 (0.66–0.88)	1.05 (0.89–1.23)	0.79 (0.68–0.90)	1.97 (1.66–2.34)
THA	No	22,595	322	1.43 (1.27–1.58)	1	0.88 (0.75–1.00)	1	0.55 (0.45–0.65)	1
	Aspirin	3,564	70	1.96 (1.51–2.42)	1.39 (1.07–1.80)	1.18 (0.82–1.53)	1.35 (0.97–1.89)	0.79 (0.50–1.08)	1.43 (0.95–2.17)
LMWH (N = 68,834)									
TKA	No	108,887	1,240	1.14 (1.08–1.20)	1	0.74 (0.69–0.79)	1	0.40 (0.36–0.44)	1
	LMWH	55,181	1,559	2.83 (2.69–2.96)	2.52 (2.34–2.72)	1.83 (1.71–1.93)	2.50 (2.28–2.74)	1.00 (0.92–1.09)	2.51 (2.22–2.85)
THA	No	22,595	322	1.43 (1.08–1.20)	1	0.88 (0.75–1.00)	1	0.55 (0.45–0.65)	1
	LMWH	13,653	355	2.60 (2.33–2.87)	1.85 (1.59–2.15)	1.55 (1.35–1.76)	1.78 (1.47–2.17)	1.05 (0.88–1.22)	1.92 (1.51–2.44)
Rivaroxaban (N = 69,702)									
TKA	No	108,887	1,240	1.14 (1.08–1.20)	1	0.74 (0.69–0.79)	1	0.40 (0.36–0.44)	1
	Rivaroxaban	64,859	531	0.82 (0.75–0.89)	0.72 (0.65–0.79)	0.51 (0.46–0.57)	0.69 (0.61–0.79)	0.31 (0.27–0.35)	0.77 (0.65–0.91)
THA	No	22,595	322	1.43 (1.27–1.58)	1	0.88 (0.75–1.00)	1	0.55 (0.45–0.65)	1
	Rivaroxaban	4,843	96	1.98 (1.59–2.37)	1.40 (1.11–1.76)	1.38 (1.05–1.71)	1.59 (1.20–2.10)	0.60 (0.38–0.82)	1.09 (0.73–1.64)
Fondaparinux (N = 8,718)									
TKA	No	108,887	1,240	1.14 (1.08–1.20)	1	0.74 (0.69–0.79)	1	0.40 (0.36–0.44)	1
	Fondaparinux	7,721	106	1.37 (1.11–1.63)	1.21 (0.99–1.48)	0.60 (0.42–0.77)	0.81 (0.60–1.09)	0.78 (0.58–0.97)	1.94 (1.48–2.55)
THA	No	22,595	322	1.43 (1.27–1.58)	1	0.88 (0.75–1.00)	1	0.55 (0.45–0.65)	1
	Fondaparinux	997	10	1.00 (0.38–1.62)	0.70 (0.37–1.32)	0.60 (0.12–1.08)	0.68 (0.30–1.55)	0.40 (0.01–0.79)	0.73 (0.27–1.98)

Because overall pharmacological prophylaxis was not associated with the reduction of postoperative VTE rates, we performed a further analysis in accordance with the pharmacologic agents used (Table 4) and the length of pharmacological prophylaxis in each surgery (Table 5). The cutoff (i.e., 10 days) was selected because it was the least requirement for adequate thromboprophylaxis following major orthopedic surgery in the ACCP guideline [1]. Comparing the postoperative VTE rates based on the pharmacologic agents used, the rates were higher in patients with pharmacological prophylaxis for most groups than in those without prophylaxis. However, rivaroxaban significantly reduced the postoperative VTE rate in patients undergoing TKA (0.82% *vs*. 1.14%; OR 0.72 [95% CI, 0.65–0.79]; Table 4). Furthermore, patients who received ≥ 10 days of pharmacological prophylaxis were significantly associated with lower rates of postoperative VTE in the TKA cohort (1.33% *vs*. 1.52%; OR 0.87 [95% CI, 0.81–0.94]; Table 5).

**Table 5:** Incidence of venous thromboembolism based on the length of pharmacological thromboprophylaxis.

Type of surgery	Length of pharmacological prophylaxis	No. of patients	No. of VTE	VTE rate (%, 95% CI)	OR (95% CI)	DVT alone rate (%, 95% CI)	OR (95% CI)	PE ± DVT rate (%, 95% CI)	OR (95% CI)
Any prophylaxis (aspirin, LMWH, rivaroxaban, fondaparinux; N = 86,501)									
Whole cohort	No or < 10 days	220,411	3,388	1.54 (1.49–1.59)	1	0.92 (0.88–0.96)	1	0.61 (0.57–0.64)	1
	≥ 10 days	86,501	1,285	1.49 (1.41–1.57)	0.97 (0.91–1.03)	0.98 (0.92–1.05)	1.07 (0.98–1.16)	0.50 (0.46–0.55)	0.83 (0.74–0.92)
TKA	No or < 10 days	182,418	2,772	1.52 (1.46–1.58)	1	0.92 (0.87–0.96)	1	0.59 (0.56–0.63)	1
	≥ 10 days	78,842	1,048	1.33 (1.25–1.41)	0.87 (0.81–0.94)	0.87 (0.81–0.94)	0.95 (0.87–1.04)	0.46 (0.41–0.51)	0.76 (0.68–0.86)
THA	No or < 10 days	37,993	616	1.62 (1.50–1.76)	1	0.96 (0.86–1.06)	1	0.67 (0.59–0.75)	1
	≥ 10 days	7,659	237	3.09 (2.71–3.51)	1.94 (1.66–2.26)	2.12 (1.80–2.47)	2.24 (1.86–2.70)	0.98 (0.77–1.23)	1.48 (1.14–1.91
Aspirin (N = 14,820)									
TKA	No or < 10 days	182,418	2,772	1.52 (1.46–1.58)	1	0.92 (0.87–0.96)	1	0.59 (0.56–0.63)	1
	≥ 10 days	12,812	212	1.66 (1.44–1.89)	1.09 (0.95–1.26)	0.99 (0.83–1.18)	1.08 (0.90–1.30)	0.66 (0.53–0.82)	1.12 (0.89–1.39)
THA	No or < 10 days	37,993	616	1.62 (1.50–1.76)	1	0.96 (0.86–1.06)	1	0.67 (0.59–0.75)	1
	≥ 10 days	2,008	39	1.94 (1.38–2.66)	1.20 (0.87–1.67)	1.59 (1.09–2.25)	1.68 (1.17–2.42)	0.65 (0.34–1.11)	0.97 (0.56–1.70)
LMWH (N = 15,730)									
TKA	No or < 10 days	182,418	2,772	1.52 (1.46–1.58)	1	0.92 (0.87–0.96)	1	0.59 (0.56–0.63)	1
	≥ 10 days	13,791	542	3.93 (3.61–4.28)	2.65 (2.41–2.91)	2.57 (2.31–2.86)	2.86 (2.55–3.21)	1.36 (1.17–1.57)	2.30 (1.97–2.69)
THA	No or < 10 days	37,993	616	1.62 (1.50–1.76)	1	0.96 (0.86–1.06)	1	0.67 (0.59–0.75)	1
	≥ 10 days	1,939	129	6.65 (5.55–7.41)	4.32 (3.56–5.26)	4.28 (3.41–5.31)	4.64 (3.64–5.91)	2.37 (1.74–3.16)	3.62 (2.64–4.98)
Rivaroxaban (N = 53,605)									
TKA	No or < 10 days	182,418	2,772	1.52 (1.46–1.58)	1	0.92 (0.87–0.96)	1	0.59 (0.56–0.63)	1
	≥ 10 days	50,085	280	0.56 (0.50–0.63)	0.36 (0.32–0.41)	0.40 (0.35–0.46)	0.44 (0.38–0.51)	0.16 (0.12–0.19)	0.26 (0.21–0.33)
THA	No or < 10 days	37,993	616	1.62 (1.50–1.76)	1	0.96 (0.86–1.06)	1	0.67 (0.59–0.75)	1
	≥ 10 days	3,520	61	1.73 (1.33–2.23)	1.07 (0.82–1.40)	1.28 (0.93–1.71)	1.34 (0.98–1.83)	0.46 (0.26–0.74)	0.68 (0.41–1.13)
Fondaparinux (N = 2,346)									
TKA	No or < 10 days	182,418	2,772	1.52 (1.46–1.58)	1	0.92 (0.87–0.96)	1	0.59 (0.56–0.63)	1
	≥ 10 days	2,154	14	0.65 (0.36–1.09)	0.42 (0.25–0.72)	0.23 (0.07–0.54)	0.25 (0.10–0.61)	0.42 (0.19–0.79)	0.70 (0.36–1.35)
THA	No or < 10 days	37,993	616	1.62 (1.50–1.76)	1	0.96 (0.86–1.06)	1	0.67 (0.59–0.75)	1
	≥ 10 days	192	2	1.04 (0.13–3.76)	0.64 (0.16–2.58)	1.04 (0.13–3.76)	1.09 (0.27–4.41)	0 (0–1.92)	

### Risk factors for postoperative VTE following TKA/THA

The results of univariate and multivariate logistic regression analyses are summarized in Table 6. In the multivariable analysis, the odds of VTE were significantly greater for patients aged ≥ 60 years (OR, 2.20; 95% CI, 1.98–2.45), male (OR, 1.23; 95% CI, 1.14–1.32), THA (OR, 1.15; 95% CI, 1.05–1.26), ≥ 10 days of prophylaxis (OR, 0.79; 95% CI, 0.74–0.85), and general anesthesia (OR, 2.62; 95% CI, 2.46–2.79).

**Table 6:** Univariate and multivariate analyses to identify independent risk factors for venous thromboembolism development in the study cohort.

		Univariate analysis		Multivariate analysis	
		OR (95% CI)	*P* value	OR (95% CI)	*P* value
Age	< 60 years (referent)	1		1	
	≥ 60 years	1.65 (1.50–1.82)	< 0.0001	2.20 (1.98–2.45)	< 0.0001
Sex	Female (referent)	1		1	
	Male	1.22 (1.14–1.31)	< 0.0001	1.23 (1.14–1.32)	< 0.0001
Surgery	TKA (referent)	1		1	
	THA	1.28 (1.19–1.38)	< 0.0001	1.15 (1.05–1.26)	0.0019
Length of prophylaxis	No or < 10 days (referent)	1		1	
	≥ 10 days	0.97 (0.91–1.03)	0.2972	0.79 (0.74–0.85)	< 0.0001
Mode of anesthesia	Regional (referent)	1		1	
	General	2.59 (2.44–2.75)	< 0.0001	2.62 (2.46–2.79)	< 0.0001
Pharmacological prophylaxis	No (referent)	1		1	
	Yes	1.20 (1.11–1.30)	< 0.0001	1.11 (0.98–1.24)	0.0583

**Fig 2:**
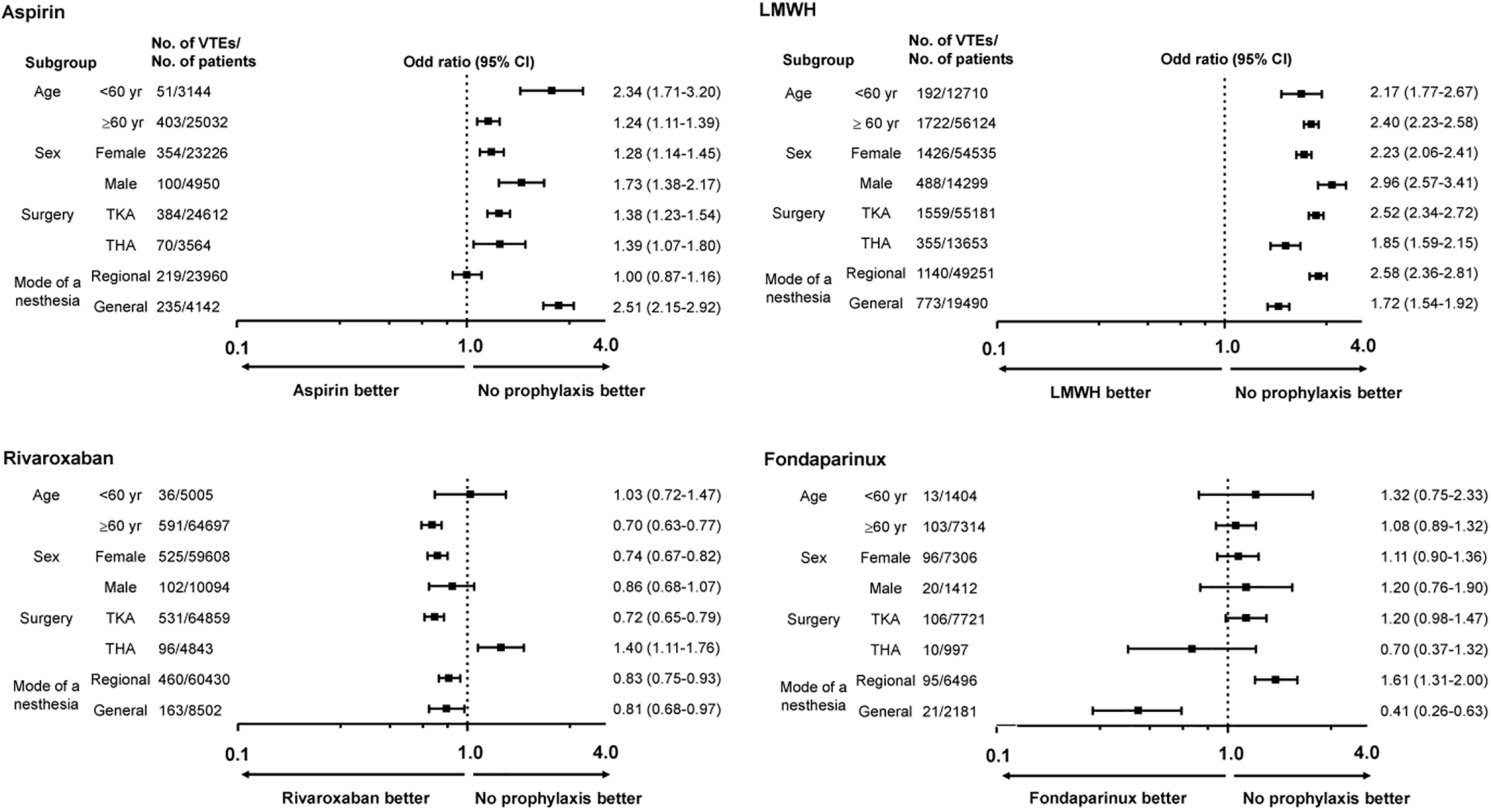
The risk of VTE in specific subgroups based on the pharmacologic agents used.

Fig 2 shows a plot of ORs (with 95% CIs) for the subgroup analyses that evaluated the benefit of each pharmacological agent with respect to clinical characteristics, which were identified as risk factors for postoperative VTE development following TKA/THA in the study. Rivaroxaban was associated with a benefit in most subgroups examined, including patients aged ≥ 60 years and those received general anesthesia (Fig 2). However, a benefit from using other drugs was not prominent in this analysis.

### Red blood cell transfusion

During the hospital stay, 258,538 patients (84.24%) were administered red blood cell transfusion with a mean of 3.21 units (95% CI, 3.20–3.22). Among patients undergoing TKA and THA, 222,048 (85.00%) and 36,454 (79.85%) patients had red blood cell transfusions (mean, 3.52 units and 3.41 units, respectively). Red blood cell transfusions were compared based on the pharmacological agents, and pharmacological prophylaxis using LMWH, rivaroxaban, and fondaparinux significantly increased the number of patients who were administered red blood cell transfusion and the amount of transfused red blood cells in patients undergoing TKA and THA (Table 7). However, aspirin did not increase red blood cell transfusion compared with no pharmacological prophylaxis in both surgeries (Table 7).

**Table 7:** Red blood cell transfusions based on the pharmacological agents used.

Type of surgery	Pharmacological prophylaxis status	No. of patients	No. of patients received red blood cell transfusion (No., %)	OR (95% CI)	Red blood cell transfusion amount (mean, 95% CI)	OR (95% CI)
TKA	No	108,891	89,821 (82.49)	1	2.79 (2.78–2.80)	1
	Aspirin	24,612	20,227 (82.18)	0.98 (0.94–1.02)	2.93 (2.90–2.96)	1.03 (1.02–1.03)
	LMWH	55,181	49,081 (88.95)	1.71 (1.66–1.76)	3.80 (3.78–3.82)	1.17 (1.16–1.17)
	Rivaroxaban	64,859	56,363 (86.90)	1.41 (1.37–1.45)	3.56 (3.54–3.58)	1.13 (1.13–1.14)
	Fondaparinux	7,721	6,592 (85.38)	1.24 (1.16–1.32)	3.20 (3.15–3.27)	1.08 (1.07–1.09)
THA	No	22,595	17,588 (77.84)	1	2.93 (2.89–2.97)	1
	Aspirin	3,564	2,672 (74.97)	0.85 (0.79–0.93)	2.81 (2.71–2.92)	0.98 (0.97–1.00)
	LMWH	13,653	11,314 (82.87)	1.38 (1.30–1.45)	3.73 (3.67–3.80)	1.07 (1.07–1.08)
	Rivaroxaban	4,843	4,075 (84.14)	1.51 (1.39–1.64)	3.08 (3.01–3.16)	1.02 (1.01–1.03)
	Fondaparinux	997	805 (80.74)	1.19 (1.02–1.40)	3.24 (3.04–3.43)	1.03 (1.01–1.05)
Total	No	131,482	107,409 (81.69)	1	2.81 (2.80–2.82)	1
	Aspirin	28,176	22,899 (81.27)	0.97 (0.94–1.01)	2.92 (2.89–2.95)	1.02 (1.00–1.03)
	LMWH	68,834	60,395 (87.74)	1.60 (1.56–1.65)	3.79 (3.76–3.81)	1.15 (1.14–1.15)
	Rivaroxaban	69,702	60,438 (86.71)	1.46 (1.42–1.50)	3.52 (3.50–3.54)	1.11 (1.11–1.12)
	Fondaparinux	8,718	7,397 (84.85)	1.25 (1.18–1.33)	3.21 (3.15–3.26)	1.07 (1.06–1.08)

## Discussion

This study is the first to represent real practice patterns and the impact of pharmacological prophylaxis on VTE development and bleeding outcomes following TKA/THA in the Korean population. In this population-based study, despite the rapid rise in the use of pharmacological prophylaxis among Koreans, the rates of pharmacological prophylaxis (overall rate, 57.16%; rates of prophylaxis ≥ 10 days, 28.18%) were still low compared with those of Western populations [11,18,19]. The substantial underuse of the pharmacological prophylactic strategies in Korean patients was primarily due to the considerably low rates of VTE after TKA/THA in Asian populations [3–6]. In the present analysis, the incidences of VTE within 3 months of TKA and THA without pharmacological thromboprophylaxis were 0.74% and 0.88%, respectively, which were lower than those in Western populations [1]. Because of the relatively low incidence of VTE, the benefits derived from pharmacological prophylaxis are expected to be small in Koreans. Therefore, it is important to determine whether pharmacological prophylactic strategies can really reduce postoperative symptomatic VTE in a low incidence population (i.e. Korean). It was noteworthy that rivaroxaban reduced the risk of postoperative VTE in our TKA cohort. Rivaroxaban is an absolutely appealing drug for VTE prevention because it can be administered orally in a fixed-dose and has a predictable drug effect [20,21]. In this study, rivaroxaban was the most commonly used drug and had the longest duration of administration among all the drugs, resulting in a high proportion of prophylaxis ≥ 10 days within the TKA cohort. Furthermore, patients who received pharmacological prophylaxis ≥ 10 days showed lower rates of VTE following TKA than those with no or < 10 days of prophylaxis. These findings suggest that there is still room for further improvement of postoperative VTE rate, even in this low incidence population. In contrast, the use of any pharmacological thromboprophylaxis carries a risk for bleeding, hematoma formation, persisting wound drainage, and infection following TKA/THA [9], which were major concerns regarding pharmacological prophylaxis of orthopedic surgeons [7,8]. In the present analysis, pharmacological prophylaxis using LMWH, rivaroxaban, and fondaparinux increased the need for red blood cell transfusions during the postoperative period, although the severity of bleeding complications were not fully evaluated because bleeding was not objectively classified according to the ISTH definition [22]. Given these observations, it might be unreasonable to provide overall pharmacological prophylaxis using these drugs to every Korean patient undergoing TKA/THA as recommended by the international guidelines [1,2]. The ultimate objective of the guidelines is to ensure the best clinical outcomes for patients; however, the current international guidelines may lead to non-adherence and suboptimal use of pharmacological prophylaxis in low incidence populations [10]. Instead, risk-based strategies with accurate identification of evidence-based risk groups for VTE are more appropriate for Korean patients undergoing TKA/THA. In addition, it is notable that aspirin did not increase the need for blood transfusion despite no clear benefit for the prevention of VTE in our analysis. Recent data also suggested the possibility that aspirin might have an influence on the prevention of postoperative arterial thrombosis after TKA/THA [23]. Considering that advanced age, male sex, and general anesthesia were identified as risk factors in this study, these parameters may be candidates for future studies to further investigate the risk-adapted thromboprophylactic strategies. Patients who do not have any risk factors might have excellent outcomes with very low rates for developing postoperative VTE. These patients may be eligible for study with low bleeding risk thromboprophylaxis strategies (i.e., aspirin, mechanical prophylaxis). In contrast, patients with risk factors for VTE should be considered in the studies of strategies incorporating standard pharmacological prophylaxis in the Western populations. Therefore, our data provide positive evidence for risk-based perioperative thromboprophylactic strategies in the Korean population. Such findings are also important for other Asian populations with similar risks because they assist in estimating both the incidence of VTE and the benefit from pharmacological thromboprophylaxis.

Interestingly, patients who received drugs for VTE prevention in this study had higher rates of VTE than those who did not receive anticoagulants. This might be due to several factors. First, the universal pharmacological prophylaxis was not generally recommended to those undergoing TKA/THA in Korea because the incidence of VTE was relatively low [3,14]. Instead, prophylactic treatments were selected based on both the surgical procedure and the VTE risk level of each patient [9,24]. In the 2^nd^ edition of the Korean VTE prevention guideline [24], mechanical or pharmacological prophylaxis was recommended to patients with moderate risk of VTE; and pharmacological prophylaxis (± mechanical prophylaxis) was recommended to patients with high risk for VTE. According to this guideline, TKA/THA is considered as a moderate risk procedure. However, when a patient has additional risk factors, including advanced age, general anesthesia, previous VTE history, or thrombophilia, TKA/THA is considered high risk for VTE. Because of this, it is likely that patients received pharmacological prophylaxis in our cohort may already have a higher risk for developing VTE compared with those who did not receive anticoagulants. Our findings support this notion. Patients with pharmacological prophylaxis had more clinical features—advanced age, longer hospital stay, and general anesthesia—posing a greater risk for VTE. Another potential explanation was the low rates of extended-duration prophylaxis in our cohort. In the present analysis, approximately 57% of patients received any kinds of prophylactic drugs, indicating that physicians realized the need for pharmacological prophylaxis in more than half of patients. However, approximately 28% of all patients received pharmacological prophylaxis ≥ 10 days; particularly, only 16% of patients undergoing THA received pharmacological prophylaxis ≥ 10 days. Previous studies have reported that the median time to the diagnosis of symptomatic VTE from surgery was 5 to 15 days for TKA and 12 to 22 days for THA [5,18,19,25,26]. Although we could not observe the time course of VTE following TKA/THA due to the lack of available data in the HIRA database, these findings still suggest that a substantial number of patients with TKA/THA, particularly THA, who required pharmacological prophylaxis might be at greater risk for VTE without the use of pharmacological prophylaxis during the postoperative period. Although the effectiveness of extended thromboprophylaxis after TKA/THA has been debated [27], Eikelboom *et al*. [28] showed the efficacy of extended-duration prophylaxis for 30–42 days using LMWH on the reduction of symptomatic VTE. Therefore, considering that the risk for VTE remains for 3-month period following the surgery [25], early discontinuation of pharmacological prophylaxis in high risk patients may attribute to the late occurrence of VTE, resulting in paradoxical high postoperative VTE rates in patients who received pharmacological prophylaxis.

This study has several potential limitations. First, the present analysis did not investigate the frequency of mechanical prophylaxis use and early mobilization strategies, because they required a review of the source medical records. Actually, the bleeding risk of patients plays a critical role in the decision to provide VTE prophylaxis and could lead to the use of mechanical thromboprophylaxis rather than anticoagulation [11]. Furthermore, a substantial proportion of patients with pharmacological prophylaxis might receive dual prophylaxis using anticoagulants and mechanical devices during hospital stay. However, from the HIRA database, it is impossible to identify whether mechanical prophylaxis has been used. Thus, we cannot assume the effect of mechanical prophylaxis on VTE following TKA/THA in our cohort. Similarly, although fast-track protocols with early mobilization might impact on the incidence of VTE after TKA/THA despite the short length of pharmacological thromboprophylaxis [29], we could not distinguish these strategies in the HIRA database because it also required the review of source medical records. Therefore, caution is needed to interpret our data. Second, because of some differences from Western countries in the real clinical practices of pharmacological thromboprophylaxis, we adopted arbitrarily predefined cutoffs for pharmacological thromboprophylaxis (i.e. anticoagulant administration within 3 days after surgery). Indeed, Korean orthopedic doctors tend to delay anticoagulant administrations until complete surgical hemostasis has been achieved. Consequently, drug administration was not infrequently delayed for a couple of days. In addition, there is a possibility that patients’ compliance is low in specific pharmacological agents within the group prescribed for > 10 days, particularly requiring parenteral injection (i.e. LMWH), which might affect our results. However, we have no way to know patients’ compliance with pharmacological prophylaxis after discharge in the HIRA database. Thus, a great caution is required to compare our data with other studies. Third, because the diagnosis of VTE was based on the information from the HIRA database, there could be discrepancies between the data from HIRA and source medical records. For instance, patients with VTE who did not receive UFH, LMWH, or therapeutic dose of rivaroxaban could not be selected as a VTE case. Actually, anticoagulants are not administered to patients with postoperative active bleeding. Patients who were diagnosed with acute PE might have died before the initiation of anticoagulation therapy, and there was no feasible way to identify PE events in patients who suddenly died after TKA/THA in this study. In addition, there were some possibilities that patients were diagnosed with VTE by clinical suspicion, not using objective diagnostic tests. Such VTEs cannot also be distinguished in the HIRA database. Fourth, because of a limited range of available personal information in the HIRA database, data on comorbidities, which were known to be risk factors for developing VTE during the postoperative period, were not included in the multivariate analysis. Nevertheless, to the best of our knowledge, our study is the first to investigate the patterns, trends, and clinical impact of pharmacological prophylaxis on VTE and bleeding complications in Korean patients undergoing TKA/THA.

In conclusion, this represents the largest epidemiologic study showing a gradual increase in the use of pharmacological prophylaxis in Korean patients undergoing TKA/THA, potentially reflecting an improvement in the awareness for VTE risk in the Korean population. In addition, although the incidence of VTE is still low without prophylaxis, this study shows a further reduction of postoperative VTE in patients undergoing TKA with adequate pharmacological prophylaxis given the requirement of increased transfusion of red blood cells. Considering these observations, the risk-stratified perioperative thromboprophylactic strategies need to be investigated separately for the low VTE incidence populations, including Koreans, and factors such as advanced age and general anesthesia should be incorporated into the decision making process to develop an appropriate and comprehensive thromboprophylactic strategy. Finally, this study provides valuable insights into pharmacological prophylaxis for VTE prevention following TKA/THA in populations with low VTE incidence.
